# The potential to characterize ecological data with terrestrial laser scanning in Harvard Forest, MA

**DOI:** 10.1098/rsfs.2017.0044

**Published:** 2018-02-16

**Authors:** D. A. Orwig, P. Boucher, I. Paynter, E. Saenz, Z. Li, C. Schaaf

**Affiliations:** 1 Harvard Forest, Harvard University, Petersham, MA, USA; 2 School for the Environment, University of Massachusetts, Boston, MA, USA; 3 Universities Space Research Association (USRA), Columbia, MD, USA

**Keywords:** forest dynamics, ForestGEO plot, Harvard Forest, hemlock woolly adelgid, terrestrial laser scanning, lidar

## Abstract

Contemporary terrestrial laser scanning (TLS) is being used widely in forest ecology applications to examine ecosystem properties at increasing spatial and temporal scales. Harvard Forest (HF) in Petersham, MA, USA, is a long-term ecological research (LTER) site, a National Ecological Observatory Network (NEON) location and contains a 35 ha plot which is part of Smithsonian Institution's Forest Global Earth Observatory (ForestGEO). The combination of long-term field plots, eddy flux towers and the detailed past historical records has made HF very appealing for a variety of remote sensing studies. Terrestrial laser scanners, including three pioneering research instruments: the Echidna Validation Instrument, the Dual-Wavelength Echidna Lidar and the Compact Biomass Lidar, have already been used both independently and in conjunction with airborne laser scanning data and forest census data to characterize forest dynamics. TLS approaches include three-dimensional reconstructions of a plot over time, establishing the impact of ice storm damage on forest canopy structure, and characterizing eastern hemlock (*Tsuga canadensis*) canopy health affected by an invasive insect, the hemlock woolly adelgid (*Adelges tsugae*). Efforts such as those deployed at HF are demonstrating the power of TLS as a tool for monitoring ecological dynamics, identifying emerging forest health issues, measuring forest biomass and capturing ecological data relevant to other disciplines. This paper highlights various aspects of the ForestGEO plot that are important to current TLS work, the potential for exchange between forest ecology and TLS, and emphasizes the strength of combining TLS data with long-term ecological field data to create emerging opportunities for scientific study.

## 1. Introduction

Harvard Forest (HF), located in central Massachusetts and acquired by Harvard University in 1907, is one of the oldest and most intensively studied forest research stations in the USA [1]. Originally a part of the Forestry Program at Harvard, it has a rich history of silvicultural and plant physiological research. HF has been continuously used as an outdoor laboratory to highlight the influence of past land-use history on current-day vegetation structure and function and on ecological processes [2]. In 1988, it became a long-term ecological research (LTER) site to apply an understanding of landscape history, modern and past forest dynamics, and projections of future changes in the regional and global environment [1].

### 1.1. Ecological data infrastructure at Harvard Forest

**Figure 1.**
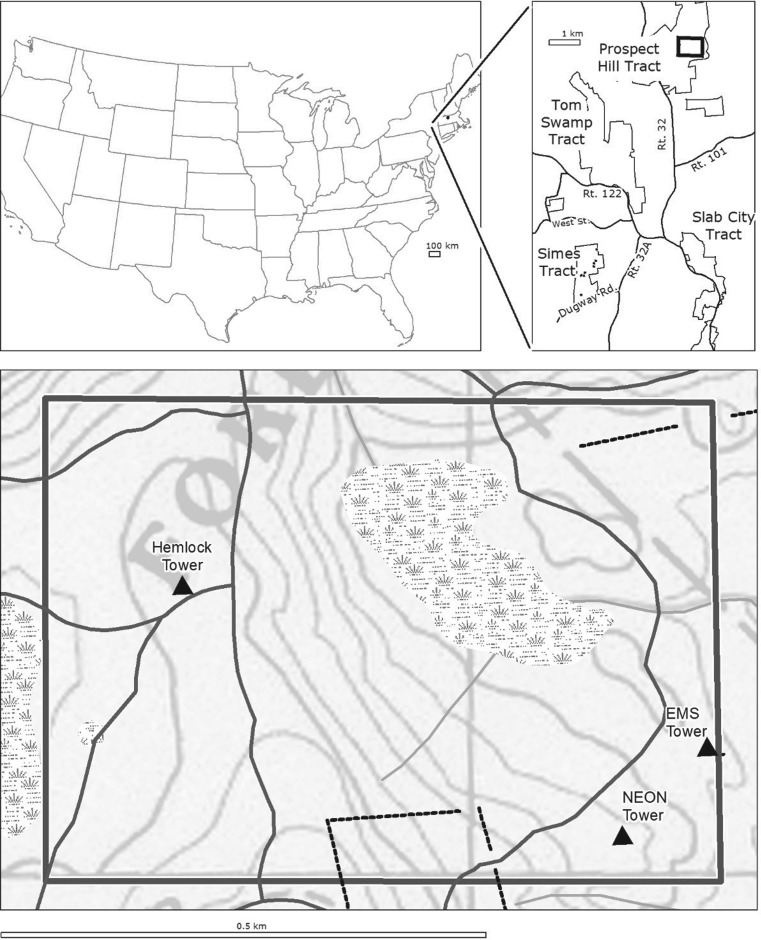
The 500×700 m ForestGEO plot located on the Prospect Hill tract of HF, showing locations of three eddy flux towers, old forest roads, stone walls (denoted by dotted lines) and the central swamp area.

As the research programme on forest ecology and change has continued to develop, extensive field infrastructure has been installed to make long-term measurements. On Prospect Hill, three eddy flux towers have been constructed: one located within a mature hemlock forest (operating since 2000), one within a mixed-hardwood forest (operating since 1989—the longest running eddy flux tower in the world) and the newly constructed National Ecological Observatory Network (NEON) tower (figure 1). A portion of the Lower Bigelow Brook watershed has also been gauged to add hydrological data to the numerous long-term research plots. With such a variety of infrastructure and over a century of field data, HF is an ideal place to test and evaluate emerging technologies for interdisciplinary collaboration.

In 2010, HF established a 35 ha Forest Global Earth Observatory (ForestGEO) plot in the area surrounding the flux towers (figure 1). Researchers have tagged, mapped and measured all woody stems ≥1 cm diameter at breast height (DBH; 1.3 m) according to standardized methods used for sampling large forest plots [3] in order to include the ForestGEO plot at HF in the global array of plots established by the Center for Tropical Forest Science and the Smithsonian Institution [4]. With repeated census activity scheduled every 5 years, the ForestGEO plot provides a field record of environmental change in a characteristic New England forest. It therefore serves as a hub for a variety of environmental research projects, such as soil analysis, biogeochemical fluxes, wildlife monitoring and forest structure, strengthening the suitability of HF as an important site for cross-comparison and transdisciplinary studies.

The ForestGEO plot also provides a baseline for documenting a major environmental change in forest structure caused by an invasive insect, the hemlock woolly adelgid (HWA; *Adelges tsugae*). Since its introduction in 1950, HWA has been killing off eastern hemlock trees (*Tsuga canadensis*) across forests in eastern North America [5–7]. Climate change is causing warming winters and enabling the insect's northward expansion into New England [8]. Hemlock is a foundation species in New England forests, serving to enforce forest structure and composition, to regulate biogeochemical cycling and to provide habitat for a variety of aquatic and terrestrial wildlife [9–12]. HF established the ForestGEO plot only 2 years after the first instance of HWA was observed in local forests (2008). The ForestGEO plot is expected to lose the majority of its hemlocks within a decade or two from the onset of infestation, allowing deciduous species to succeed and establish a new forest composition. The resampling of the ForestGEO plot is therefore serving to monitor the progressive impact of HWA and characterize the associated changes in forest composition. Mortality assessments of the ForestGEO plot, such as that conducted in the summer of 2016 (described below), are aiding in monitoring the impact of HWA.

### 1.2. Remote sensing data at Harvard Forest

The combination of long-term field plots, eddy flux towers and the detailed past history of the ForestGEO site has made HF very appealing for a variety of remote sensing studies. For example, a number of studies have used various satellite applications to characterize forest biophysical and structural parameters, including a comparison of MODerate Resolution Imaging Spectroradiometer (MODIS) satellite-derived and flux-tower-derived land surface albedo [13] and an estimation of forest canopy structure and composition from an Airborne Visible/Infrared Imaging Spectrophotometer (AVIRIS) imaging spectrometer combined with Land, Vegetation, and Ice Sensor (LVIS) waveform lidar measurements to improve model simulations of ecosystem function [14]. In addition, LVIS lidar was used to assess canopy structure over 65 years following the 1938 hurricane [15]. Field-measured aboveground biomass (AGB) in forested plots has been compared with estimates of forest biomass derived from L-band radar backscatter observed from the NASA/Jet Propulsion Laboratory (JPL) Uninhabited Aerial Vehicle Synthetic Aperture Radar (UAVSAR) instrument [16] as well as from a combination of NASA's airborne LVIS and the ground-based, hemispherical-scanning, near-infrared lidar Echidna^®^ Validation Instrument [17–19]. Furthermore, as part of the NEON standardized aquatic and terrestrial sampling [20], an aerial observation platform has been used to conduct periodic surveys with a shortwave infrared imaging spectrometer, a scanning small-footprint waveform lidar and a high-resolution airborne digital camera [21].

The emerging technology of using terrestrial laser scanning (TLS) has been applied to the well-established sites in the HF to test and validate its capabilities to improve forest structure measurements and to discover and answer new questions on forest ecology. Terrestrial laser scanners are ground-based active remote sensing instruments that emit pulses of light at a specific set of wavelengths and record the returning energy that is reflected off objects in the space surrounding the instrument. TLS analyses this reflected light to generate a series of discrete points that outline the objects intercepted by the emitted pulses. TLS data are commonly referred to as a point cloud since the data generate a series of points with coordinates in three-dimensional (3D) space. Thus, the result of each terrestrial laser scan is a sample of the structural and reflective properties of the 3D space and the objects that surround the instrument.

TLS technology is relatively new, and, while there are a variety of commercial TLS instruments available for purchase, many researchers choose to design and build their instruments in house. One such instrument used extensively at HF is the Compact Biomass Lidar (CBL). The CBL instrument design is based on an initial concept by the Katholieke Universiteit Leuven [22], realized by the Rochester Institute of Technology [23], and extensively refined (and currently produced in a scientific mode) by the University of Massachusetts, Boston, MA, USA [24]. The CBL makes use of a commercially available lidar (the SICK LMS-151), a custom-built rotary stage and on-board data processing to produce a robust, lightweight, rapid scanning instrument that has already been deployed over a wide range of environments [23–25]. The CBL's portability and scan speed have been put to use in HF to sample hundreds of 20 × 20 m plots in the ForestGEO plot within a matter of days. The speed of the CBL, and of TLS technology in general, is serving as a major advantage for ecology, bolstering traditional forestry methods of plot census and disturbance monitoring.

### 1.3. Potential to combine forest ecology and terrestrial laser scanning research

As advances in lidar technology and data availability have improved [26], additional ground-based TLS studies have been conducted to characterize vegetation structure at the site and plot scale in the HF ForestGEO plot. We highlight the various aspects of the ForestGEO plot that are important to current TLS work and future ecological applications of TLS. Our objective in this review paper is to introduce examples of ongoing research work employing TLS applications to forest ecosystems at HF, describe their contributions to our understanding of the forest and discuss emerging opportunities to use TLS technology in a variety of ecological applications. In this way, the exchange of knowledge between these two scientific fields will be examined in order to illustrate the strengths of drawing dually from ecological and TLS data. We also provide a framework for how TLS sampling can be improved upon in the future.

## 2. Study area

HF is located in Petersham, in north-central Massachusetts, USA. The Prospect Hill Tract is located on the northern, highest portion of the major ridge in Petersham, and contains the 35 ha ForestGEO plot (figure 1). The plot dimensions are 500 m to the north and 700 m to the east from the southwest corner (35 ha; 42.54°N, 72.18°W). Elevations in the plot range from 340.2 to 367.8 m.a.s.l. Soils are classified as either Pillsbury–Peacham, Becket–Skerry or Peru–Marlow association. These are gravelly and fine sandy loam soils that developed in glacial tills overlying gneiss and schist bedrock (NRCS n.d.). The north-central portion of the plot contains a 3 ha swamp section containing a peat bog and muck soils that have been affected by various levels of beaver activity over the last century. The plot is located within the Worcester/Monadnock Plateau ecoregion [27] and is characterized by an average annual temperature of 7.1°C and annual precipitation of 106 cm, distributed evenly throughout the year [28]. Regional vegetation is characterized as transition hardwoods–white pine–hemlock [29]. The HWA was first observed on eastern hemlock in the ForestGEO plot in 2008, and is now widely distributed throughout the plot (DA Orwig 2008, personal observation). HWA was accidentally introduced from Japan into Virginia during the 1950s [30], and the insect has spread northeast over the ensuing 50 years into New England [31], leading to widespread hemlock decline and mortality [7].

The ForestGEO location has served as a hub of research activity for decades (summarized in [32]). In addition to flux measurements from the towers, a wide array of additional measurements have occurred within the plot, including ecosystem (litterfall, soil respiration, soil nitrogen availability, hydrology, canopy phenology), microenvironmental (photosynthetically active radiation, leaf area index (LAI), soil moisture and temperature) and forest dynamics (dendrometer bands, tree core analyses, understorey vegetation and long-term hemlock history). These data have then served as input to various forest modelling efforts including the Ecosystem Demography, SORTIE, LANDIS and RHESSys hydrological models.

## 3. Discussion

This paper examines the exchange between TLS and forest ecology in three sections. The first section discusses ongoing work with TLS at HF, with a few case studies illustrating how current TLS technologies have benefited from the data resources at HF and vice versa. A second section builds on the first to discuss emerging opportunities for TLS study at HF given the challenges to current studies, including the potential to incorporate TLS studies into the LTER infrastructure already present at HF. Due to rapid advances in TLS technology and increased use of TLS instruments, there is a need to refine TLS sampling strategies for ecological applications. We provide a conceptual framework for improving sampling scheme design in the third section. We conclude this paper by highlighting the exchange of knowledge occurring between TLS and ecological data at HF.

### 3.1. Ongoing terrestrial laser scanning work at Harvard Forest

In the process of reviewing the variety of TLS work in the ForestGEO plot at HF, three general categories of TLS studies have emerged: studies that have employed TLS to develop and measure forest parameters, studies that aim to improve TLS observations by combining with other remote sensing technologies for a multi-angle view of the forest, and, finally, studies that have used TLS to characterize forest type and condition. These three categories are outlined with case studies of published work from HF.

#### 3.1.1. Forest structure measurements

Over the past decade, ground-based Echidna lidar scans have been used to estimate mean DBH values of trees, stem density, basal area and woody biomass [18], and effective leaf area index, foliage profiles and stand height [19]. Additional information has been gained from creating 3D forest reconstructions, including retrieving canopy structural parameters [33] (data available at Oak Ridge National Laboratory) [34]. By examining scans over time, TLS was able to document the impact of ice storm damage on the HF, including reductions in LAI and stem density and increases in the mean DBH of trees as the smaller, weakened trees were felled by the storm [35]. These studies add to the growing body of work elsewhere measuring forest structural elements [36–39].

A recent advance in TLS technology has enabled new ways of quantifying photosynthetic activity in forests by integrating multiple lasers at different wavelengths to collect multispectral scans. One such research instrument, the Dual-Wavelength Echidna Lidar (DWEL), has been deployed at several sites at the HF, including hardwood-dominated and hemlock-dominated plots in the ForestGEO region. The DWEL uses two lasers pulsing simultaneously at two different wavelengths of 1064 and 1548 nm. At the 1548 nm wavelength, laser power returned from leaves is much lower than from woody materials, such as trunks and branches, due to absorption by liquid water in leaves. In contrast, returned power from both leaves and woody materials is similar at the other wavelength of 1064 nm [40–42]. Such a contrast in the spectral responses of leaves and woody materials has been used to produce a 3D classification of the primary photosynthetic component of forests (leaves) and the non-photosynthetic component (woody materials) [43]. A similar instrument (Salford Advanced Laser Canopy Analyzer [44,45]) has also recently been deployed at HF.

In general, ongoing work with TLS on forest structural metrics has contributed to the TLS field, establishing the technology as a means of developing new metrics and providing a means for direct quantification of parameters that previously had been obtained through extensive fieldwork, or predicted from other remote sensing data at coarse scales (LAI, for instance). A recent US National Science Foundation/Research Coordination Network-funded [46] field campaign at HF during August 2017 deployed seven TLS instruments to scan a 50 × 50 m hemlock–hardwood plot, and paired this activity with destructive sampling of four tree species (20 trees total) to compare biomass estimations and structural measurements across different TLS instruments. It is not a necessity to have a field site such as HF to evaluate these new structural metrics, but lidar deployments at other ForestGEO sites (such as Wytham Woods, UK [47]) have shown that the existing forestry data and infrastructure, such as that of the tagged and mapped trees in the ForestGEO plot, lend themselves to further studies seeking to refine TLS-derived forest metrics.

For instance, plot data measurements of canopy structure have been combined with high spatial resolution airborne lidar from the Goddard LiDAR, Hyperspectral, and Thermal sensor package (G-LiHT) [48] to validate field-based crown geometry allometric equations. This data combination yielded a simple canopy height model and a two-dimensional canopy height raster of the field-based stem map [49]. TLS data were also collected from within the plot and are being compared with airborne lidar and the canopy allometric models.

#### 3.1.2. Data fusion and multi-angle observations to improve terrestrial laser scanning measurements

A major challenge for TLS studies in dense forests is occlusion from understorey and mid-storey branches that prevent scanners from collecting reliable information from the top of canopies. One solution to this issue has been the combination of airborne laser scanning (ALS) and TLS data together, combining information about the canopy from above and below [50]. Terrestrial laser scans have been successfully combined with ALS data to measure traditional forest parameters, such as AGB [17]. In addition, discrete TLS and ALS data over HF have been combined to generate additional forest parameters, such as canopy density and thickness [50], and rugosity [51]. Combining terrestrial and airborne remote sensing platforms unveils new possibilities for forest quantification, since the combined information of multiple sensors and view angles allows the 3D space of the forest to be recorded with high information density.

Additional opportunities to merge airborne with terrestrial lidar are possible in the future with a plan for annual acquisition by the NEON Airborne Observation Platform [21] which includes discrete and waveform lidar data, hyperspectral data and red/green/blue (RGB) imagery. These airborne data offer the possibility of characterizing fine-scale forest structure, canopy characteristics and annual changes associated with natural disturbance and successional dynamics.

While airborne lidar observations can help reduce issues of occlusion, they are only collected infrequently (often only one data collection per year), and the point density for discrete airborne observations is much lower than for TLS. Rapid-scanning TLS instruments, however, can capture high-resolution canopy structure when raised into the canopy. A higher resolution dataset can be obtained by deploying TLS instruments at various height levels, whether by raising the instrument up on a tripod [50], climbing a tower or riding up in a bucket lift—as was facilitated during the recent TLS field campaign at HF.

#### 3.1.3. Evaluating forest type and condition at the plot scale

TLS observations in HF have been used to demonstrate the ability of lidar instruments to be sensitive to the ecosystem type and condition of the scanned space. CBL scans from HF have shown promise in separating distinctly different forest structures by using the method of metaproperties analysis to distil aggregate statistics from the geometry and pulse properties of the data [25]. Furthermore, expensive, high-resolution scanners are not necessary for such scan-based techniques. In fact, laser scanners with lower point density that scan more rapidly are the best candidates for such forest classification methods, as they are able to scan more plots and cover more ground within the campaign time allotted. Such new methods of classification may be useful in delineation of habitat for conservation and ecology and for monitoring forest condition.

**Figure 2.**
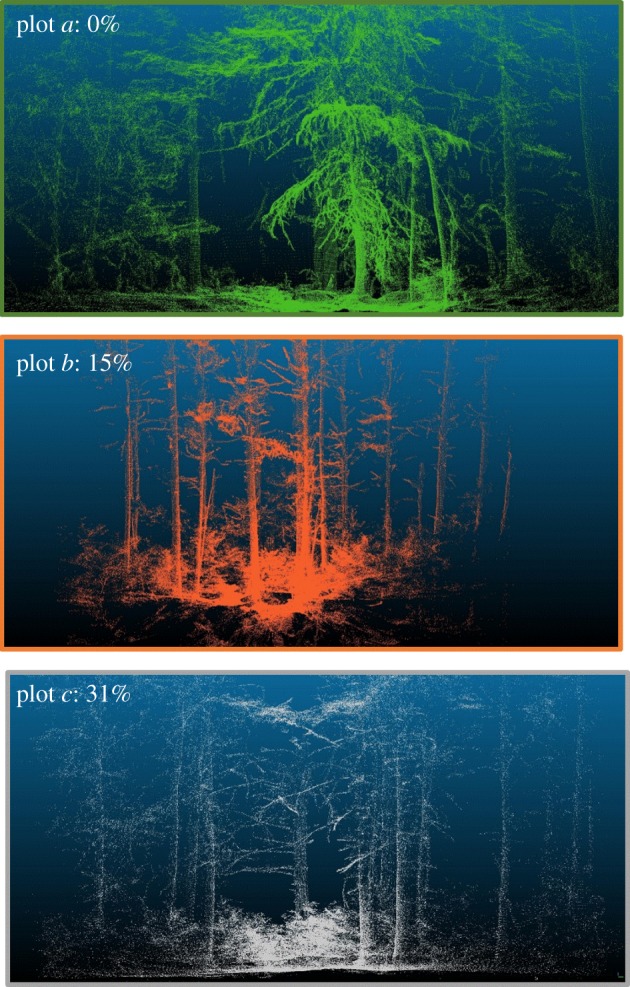
CBL scans of progressive mortality in the HF ForestGEO plot. (*a*) 0% hemlock mortality: intact branches distributed from the canopy to the ground with needles. (*b*) 15% hemlock mortality: loss of needles on lower branches and beginning of understorey vegetation establishment. (*c*) 31% hemlock mortality: branches bare, canopy damage and new understorey growing in the canopy gaps. Note: Each scan is taken from the centre of a 20×20 m plot, colour-coded and labelled here as *a*, *b* and *c* and seen on figure 3. Scans taken during summer 2016 and rendered using CloudCompare (www.cloudcompare.org).

The insect infestation at HF has also provided an ideal opportunity to test the ability of lidar to capture progressive ecosystem change in scan data. An initial study has proved successful in applying metaproperties analysis to classify infested areas of forest from healthy areas (figure 2; [25]). Research is currently ongoing to classify the progressive stages of the infestation using the CBL [24], a lightweight and highly portable terrestrial lidar scanner.

### 3.2. Emerging opportunities with terrestrial laser scanning at Harvard Forest

#### 3.2.1. Evaluating forest health and quantifying structural change

**Figure 3.**
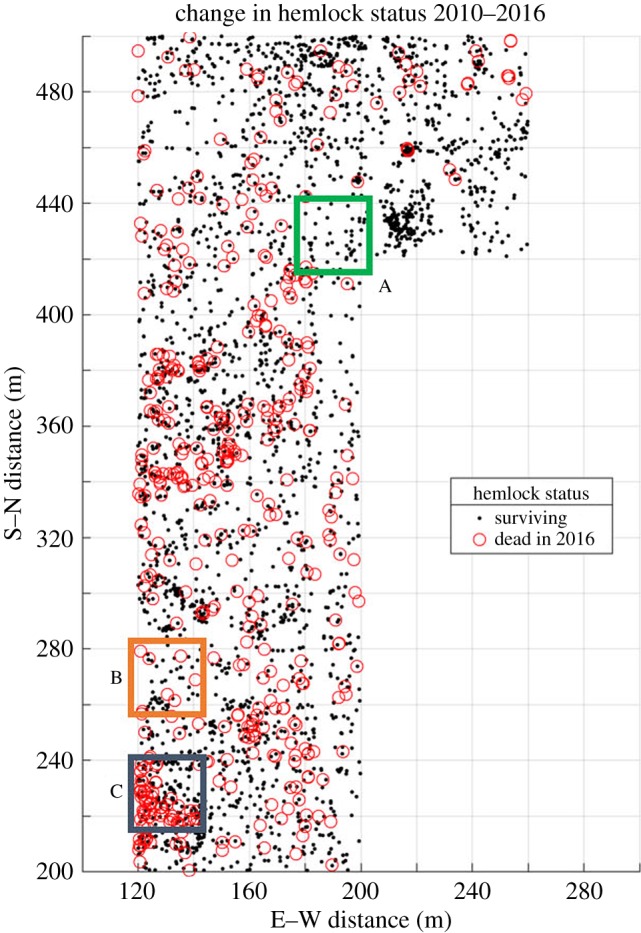
Results of the 2016 hemlock mortality assessment within 4 ha of the 35 ha HF ForestGEO plot. Although dead hemlocks were recorded from throughout the study area, there were concentrated areas of mortality in the southwest corner and middle portion of the study area. Over 60% of smaller stems (less than 10 cm DBH) died in the area although larger trees also died, including two over 75 cm DBH.

Ongoing work with TLS in HF, especially studies related to monitoring of forest condition, depend upon the continuation of field data collection efforts. The initial forest census of the entire ForestGEO plot contained over 108 000 live stems, including over 22 900 eastern hemlock stems concentrated in the western and northern portions of the plot [52]. Initial concerns of widespread hemlock mortality due to the insect infestation were validated during the summer of 2016, when a subset of hemlock-dominated quadrats (*n* = 102; totalling just over 4 ha in area) were assessed for hemlock mortality. Since 2010, 435 additional hemlock stems have died within this region, for a total of 723 stems, which now represents 20% of hemlock stems in this region of the plot (figure 3).

ForestGEO plot field data can also help TLS overcome the challenges of change detection in complex ecological environments. A TLS study that aims to map the spatial distribution of HWA-induced tree mortality at a plot scale must isolate spatial trends in mortality while excluding the spatial trends of correlated variables. TLS measurements of forest condition need to be sensitive to the progression of a disturbance (figure 2) as well identifying structural metrics (tree age, size, density, etc.) that might influence the TLS-derived plot-level mortality. For example, if smaller trees are more likely to succumb to the infestation and a TLS model is trained and tested upon an area to predict the mortality in a plot, special care must be taken to make sure that TLS estimates of plot mortality can isolate a mortality measurement that does not depend on the size of surrounding trees. TLS-derived condition and type predictions need to adapt to forest structural conditions, since even homogeneous forests have a wide variety of stem densities, tree species and other landscape features that might influence a TLS-derived measurement. Forest inventory data and mortality assessment allow for preliminary analysis to investigate spatial trends in data in order to identify and remove them from TLS-based analyses of forest condition. Thus, the combination of TLS plot-scale measurements of forest health with field data allows for the isolation of spatial trends in forest structure and condition, and can begin to identify the drivers behind the spatial distribution.

#### 3.2.2. Improving temporal resolution of ecological studies

While the data collected from large-scale, long-term ecological plots are invaluable, reassessing and monitoring these plots requires significant investments of time, funding and personnel. For that reason, reassessments often occur 5 years apart. Airborne lidar acquisition can provide annual snapshots of structural change over time, yet most study sites rarely get more than one airborne data acquisition during a year. A single portable or *in situ* TLS instrument, however, could easily improve the temporal resolution of a long-term study of forest structure, monitoring seasonal change [53], loss of stems and foliage following disturbances, tree growth and a wide variety of other structural changes in forests [38].

Establishing long-term TLS observation points within long-term field sites can greatly improve the temporal resolution of ecological field observations. The mobility and cost of TLS allows for the repeated capture of structural changes over time. Seasonal, monthly or even daily measurements are possible with automated *in situ* sensors, such as the VEGNET scanner [54,55].

Long-term monitoring with TLS also has the potential to quantify progressive structural change following disturbances. Long-term study plots are currently being set up at HF to monitor the impacts of the HWA infestation (figure 2). As HWA causes hemlock trees to lose their needles and, subsequently, their lower branches (figure 2*b*,*c*), gaps open up in the forest canopy allowing understorey to grow (figure 2*c*). During the early years of infestation, these changes are often subtle and may not be quantifiable by a human observer. With consistent monitoring of infested and control plots at the same scan angles and positions, TLS can potentially detect the onset of infestation before it becomes obvious to human observers, quantify needle and branch loss, and characterize understorey response as new tree species establish themselves in the gaps in the canopy left by defoliated and dead hemlocks.

Studies of progressive forest change are possible at sites where ecological data are bountiful and freely available. Long-term monitoring of the HWA infestation in the HF ForestGEO plot, for example, is only made possible by the efforts of ecologists and field-hands, first working to tag and record all stems over 1 cm in a 35 ha plot from 2010 to 2013, and further reassessing hemlock status (dead/alive) in a section of the ForestGEO plot in 2016 (figure 3). These forest field data are necessary for establishing baseline conditions of forest structure to calibrate TLS measurements of forest health. A full re-census of the ForestGEO plot, planned to begin in 2018, will provide an invaluable second time point for TLS studies of progressive structural change.

### 3.3. Conceptual framework for improving sampling scheme design

The rapid advance of TLS technology has resulted in improvements in the retrieval of many ecologically important variables from forest ecosystems. However, while the specifications of TLS instruments have improved iteratively, deployment strategies have remained largely untouched, typically involving the placement of TLS according to a rigid grid of a predetermined size and resolution. To fully leverage the enhanced capabilities of TLS, as well as the diversification of the technology to include more lightweight scanners, mobile scanners and flexible deployment platforms, sampling strategies are now a prominent target for refinement. Many TLS practitioners are seeking to investigate the appropriate parametrization of rigid sampling strategies for different ecological variables, instrument specifications and ecosystem structural scenarios. Additionally, the increasing power of mobile computing technology may eventually enable the quality of TLS observations to be assessed in the field, with sampling strategies altered or designed on-the-fly to improve data completeness and quality.

**Figure 4.**
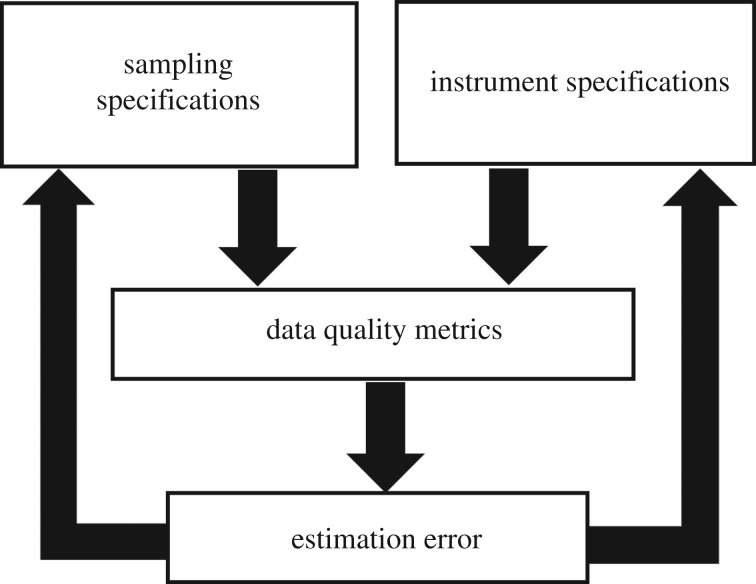
Conceptual framework for the investigation and improvement of TLS sampling scheme design and parameters on the error observed in estimation of ecological variables of interest.

To enable improvements in sampling scheme design and parametrization, the attributes of TLS sampling schemes must be linked to the error observed downstream, in the retrieval of ecological variables of interest (figure 4). This process requires several components. First, there must be validation measurements for the ecological variable, to provide quantified error as a response function. Second, there must be one or more quantifiable metrics of data quality, such as the density of observations in each region of the sample, which are retrievable from TLS observations. Intuitively, appropriate data quality metrics would have a confirmed and consistent relationship with the observed error in the ecological variable of interest. Finally, repeated and iterative experimentation with different forms and parametrizations of sampling schemes must be possible, so that the error observed downstream can inform changes to strategies upstream, and the potential improvements assessed (figure 4). It should be noted that it will always be useful to have robust characterization of the specifications and behaviour of the TLS instrument used, since the strengths and limitations of the instrument can guide more efficient improvements to sampling schemes.

The HF ForestGEO plot is an ideal study site for TLS practitioners to carry out investigations aimed at improving sampling schemes. There are a variety of sub-regions in relatively close proximity, representing a range of dominant tree morphologies, structural scenarios and even progressive stages of parasitic infestation. The complete documentation of the trees, including stem locations, stem density, DBH and samples of height and crown size, can directly provide the required validation, when these are the ecological variables of interest. In addition, the availability of stem density, size distributions and species composition provides the opportunity to investigate sampling schemes across a range of forest structure scenarios, including the ability to form stratified random sampling schemes *a priori* to increase confidence in the results. The accessibility of the site enables the repeated acquisitions necessary to iteratively refine sampling strategies, as well as test their effectiveness across changing seasons.

## 4. Summary

HF has been used at all systematic levels of TLS research, from instrument design, validation, algorithm development and testing to emerging ecological applications. HF TLS studies have contributed to ecological research in several broad ways:

(1) TLS has produced measurements of forest structure characteristics with improved efficiency and speed, allowing for wider scale and more frequent ecological observations.
(2) TLS has generated new forest parameters to quantitatively describe obscured features, such as canopy structures, by coordinating with airborne observations, space-borne remote sensing and forestry field data.
(3) TLS has characterized the condition of forest plots and has the ability to identify forest type. These instruments are valuable tools for monitoring forest health with high temporal resolution, long-term observations.

In turn, HF field studies coordinated with TLS studies have contributed and can contribute in the future to the TLS field. The useful array of infrastructure already present at HF, including eddy flux towers, terrestrial and aerial lidar acquisitions, and long-term field plots, has provided growing opportunities for ecological TLS applications:

(1) ForestGEO plot data provide identifiable plots, locations and forest census data that allow for validation of TLS-derived forest metrics and for a quantification of error in TLS sampling (figure 4).
(2) Tree mortality assessment data following the HWA infestation provide baseline data for plot-scale measurements of forest health, allowing for TLS measurements to be finely attuned to structural changes due to this progressive condition (figures 2 and 3).
(3) HF provides an arena for calibration and validation of instrument measurements. The presence of the 35 ha ForestGEO plot with locations and characteristics of every woody stem ≥1 cm DBH, and a continuous 10×10 m grid, which is recommended for a variety of TLS sampling protocols [47], provides more accurate calibration data for the characterization of forest structure and composition than smaller plots typically used for that purpose [56]. HF has also been used to calibrate and validate TLS software. For example, one study employed scan data from HF to validate an algorithm that automatically registers pairs of point clouds together that are overlapping in geographical space [57].

This exchange of knowledge has contributed widely to both fields, yet there are a variety of emerging opportunities to synthesize ecological and TLS data that were not fully covered in this paper. With the eddy flux tower infrastructure in place at HF, future work to better characterize the forest structure surrounding towers could refine the factors influencing the fluxes of water and CO_2_ measured at the towers. In addition, there are growing opportunities to integrate TLS technology with airborne and space-borne observation data to better characterize forest structure and composition. These studies linking ecological function to structure are a new frontier for collaborative research.

Finally, with the growing use of various TLS technologies of different capacities, there is a need for instrument inter-comparisons and field calibrations at well-documented test locations. These calibration activities will be essential in developing consistent measurements, evaluating TLS error and developing forest parameters. A recent example of this kind of collaborative work involved a two-week calibration activity that occurred during the summer 2017 TLS field campaign at HF.

The use of TLS and development of applications at HF has increased over the last several years and appears to be a promising tool to characterize various forest structural parameters, including estimates of tree height, biomass and crown geometry, as well as documenting dynamics associated with insect and pathogen outbreaks, natural disturbances and land-use change. The HF ForestGEO plot provides a consistent flow of ecological data that future TLS research can harness, with stem surveys occurring every 5 years, mortality assessments, constant recording of flux data, intermittent ecological and geological studies, and periodic airborne remote sensing campaigns. This wealth of data makes the ForestGEO plot unique among study sites across the globe, serving as an ideal place for TLS researchers to explore new ecological questions with emerging technology.

## Data accessibility

Additional data are available from: http://harvardforest.fas.harvard.edu:8080/exist/apps/datasets/showData.html?id=hf253.
